# Calcium-Dependent Protein Kinase 28 Maintains Potato Photosynthesis and Its Tolerance under Water Deficiency and Osmotic Stress

**DOI:** 10.3390/ijms23158795

**Published:** 2022-08-08

**Authors:** Xi Zhu, Fangfang Wang, Shigui Li, Ya Feng, Jiangwei Yang, Ning Zhang, Huaijun Si

**Affiliations:** 1 State Key Laboratory of Aridland Crop Science, Gansu Agricultural University, Lanzhou 730070, China; 2 South Subtropical Crops Research Institute, Chinese Academy of Tropical Agricultural Sciences, Zhanjiang 524091, China; 3 College of Life Science and Technology, Gansu Agricultural University, Lanzhou 730070, China

**Keywords:** *StCDPK28*, drought stress, osmotic stress, potato

## Abstract

Calcium-dependent protein kinases (CDPK) are implicated in signaling transduction in eukaryotic organisms. It is largely unknown whether *StCDPK28* plays a role in the response to water deficiency and osmotic stress in potato plants (*Solanum tuberosum* L.). Potato cv. Zihuabai was cultivated under natural, moderate, and severe water deficiency conditions; to induce osmotic stress, potato plants were treated with 10% or 20% PEG. *StCDPK28*-overexpression and *StCDPK28*-knockdown plants were constructed. *StCDPKs* were evaluated by qRT-PCR. The subcellular location of the StCDPK28 protein was observed with confocal scanning laser microscopy. Phenotypic changes were indicated by photosynthetic activity, the contents of H_2_O_2_, MDA and proline, and the activities of CAT, SOD and POD. Results showed water deficiency and osmotic stress altered *StCDPK* expression patterns. StCDPK28 exhibited a membrane, cytosolic and nuclear localization. Water deficiency and osmotic stress induced *StCDPK28* upregulation. Photosynthetic activity was enhanced by *StCDPK28* overexpression, while decreased by *StCDPK2* knockdown under water deficiency and osmotic stress. *StCDPK28* overexpression decreased H_2_O_2_ and MDA, and increased proline, while *StCDPK28* knockdown showed reverse results, compared with the wild type, in response to water deficiency and osmotic stress. *StCDPK28* overexpression increased the activities of CAT, SOD and POD, while *StCDPK28*-knockdown plants indicated the reverse trend under water deficiency and osmotic stress conditions. Regulation of StCDPK28 expression could be a promising approach to improve the tolerance ability of potato plants in response to drought or high salt media.

## 1. Introduction

Potato (*Solanum tuberosum* L.) is a staple and important food for populations in developing countries, particularly in the temperate region of northern China [1]. Unfortunately, potato plants are exposed to several environmental stressors that restrict their growth, development and harvest [2]. Drought is an important adverse factor for plant cultivation, and water deficiency affects the entire life cycle of the potato plant, such as tuber formation [3]. Nowadays, water deficiency is a growing conundrum in the context of global climate change [4]. As another limiting factor, osmotic stress causes a loss of approximately 20–98% of potato production [5]. Consequently, high priority should be given to enhancing the transient drought tolerance of potato plants based on conventional breeding and biotechnology. The development of transgenic potato plants enhances the drought and salt stress tolerance and increases tuber yield [6]. Calcium-dependent protein kinases (CDPK) are identified as crucial modulators for signaling transduction in eukaryotic organisms [7], which can be used as potential targets for biotechnology.

Protein kinases are a class of protein structural domains consisting of 250–300 amino acids that are responsible for phosphate transfer reactions. Emerging data indicate that plants evolve protein kinases to respond to stress conditions such as waster deficiency and osmotic stress [8,9,10]. CDPKs are characteristic of a calmodulin-like domain with EF-hand Ca^2+^-binding sites, a junction domain, a typical Ser/Thr protein kinase domain, and a variable N-terminal domain [11]. Ca^2+^ is a second messenger in the molecular regulatory mechanism and it is involved in the activation of CDPKs [12]. Multiple stimuli, such as water deficiency and osmotic stress, induce changes in the cellular Ca^2+^ concentration and subsequent activation of CDPKs, triggering the binding of Ca^2+^ with the calmodulin-like domain [13,14]. Downstream targets are continually modulated in plants, and numerous physiological processes are changed in response to biotic stressors [13,14]. In potato plants, 26 StCDPKs have been predicted and classified into four groups (subfamilies I, II, III and IV) [15]. Their functions have been successively elucidated. For example, StCDPK1 has been shown to play a role in tuber transition and sprouting by affecting the vascular system of stems and roots [16].

CDPK28 is one of the calcium-dependent protein kinases that has been extensively studied in *Arabidopsis thaliana* [17,18,19,20,21,22]. The regulatory function of CDPK28 at the phenotypic level has been increasingly revealed. AtCDPK28 was found to regulate hypocotyl and lignification phenotypes, which is attributed to phosphorylation in methionine adenosyltransferases that affect lignin deposition and ethylene biosynthesis [17]. Further, CDPK28 is reported to mediate the sodium-chloride- and mannitol-sensitive phenotypes in *Arabidopsis* [23]. In terms of the molecular mechanism, CDPK28 has been proven to have a high affinity Ca^2+^/CaM-binding protein, and the peptide kinase activity of CDPK28 is associated with its autophosphorylation in response to the Ca^2+^ messenger [18]. In the context of biotic stressors, CDPK28 modulates defense signaling related to reactive oxygen species and shows activity in balancing phytohormones [19]. Ding et al. recently elucidated that plasma-membrane-localized CDPK28 senses cold-induced Ca^2+^ signals and relays them to the nucleus [24]. However, it is still unknown whether StCDPK28 plays a role in the response to water deficiency and osmotic stress in potato plants.

To explore whether *StCDPK28* has functions under water deficiency and osmotic stress, we established *StCDPK28*-overexpressing and *StCDPK28*-knockdown plants and applied water deficiency and osmotic stress treatment. The result showed that *StCDPK28* was sensitive to water deficiency and osmotic stress. *StCDPK28* fortified the activity of potato in response to water deficiency and osmotic stress.

## 2. Results

### 2.1. Expression Profiles of StCDPKs in Potato Plants under Water Deficiency and Osmotic Stress, and Analysis of CDPK28 Protein

**Figure 1:**
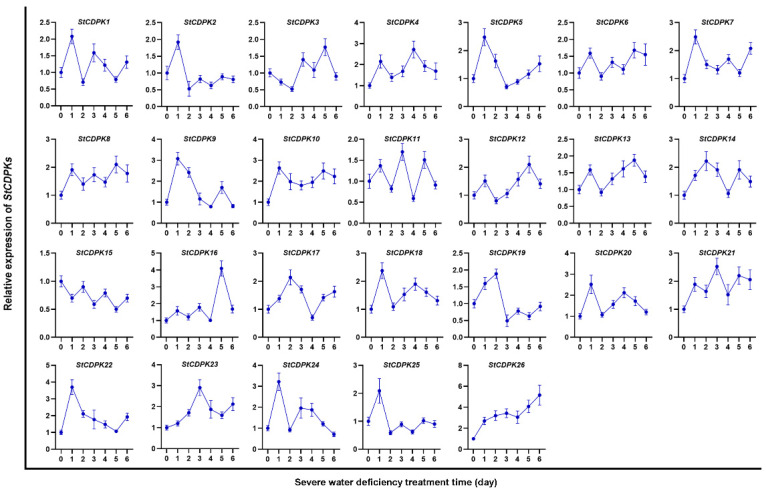
Expression profiles of *StCDPKs* in potato plants cultivated under severe water deficiency conditions. The mRNA expression of *StCDPKs* was relatively quantified by qRT-PCR and normalized to *StEf1a*. Data are presented as the means ± standard deviation of nine replicates.

**Figure 2:**
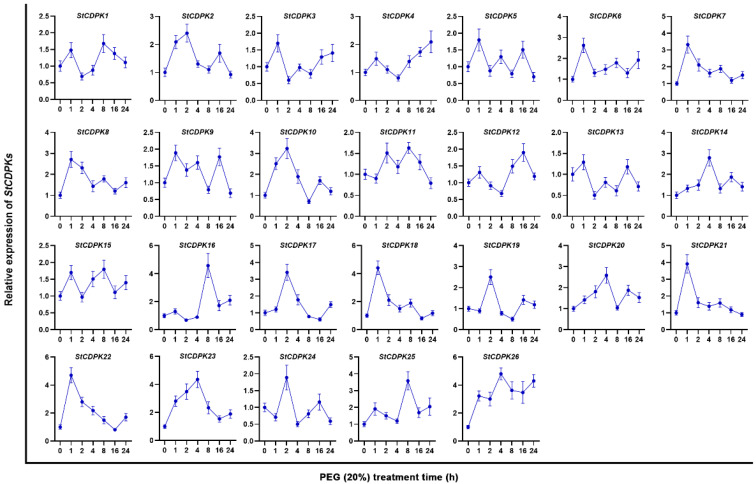
Expression profiles of *StCDPKs* in potato plants in response to 20% PEG6000. The mRNA expression of *StCDPKs* was relatively quantified by qRT-PCR and normalized to *StEf1a*. Data are presented as the means ± standard deviation of nine replicates.

Potato plants were cultivated under severe water deficiency and osmotic stress conditions, and StCDPKs were relatively quantified by qRT-PCR. Figure 1 and Figure 2 showed that water deficiency and osmotic stress changed the expression patterns of *StCDPKs*. It was worth noting that *StCDPK28* (reported as *StCDPK26* by Frantino et al.) was constantly transcribed with the extension of the incubation time under water deficiency or osmotic stress conditions. Therefore, StCDPK28 was considered to mediate potato tolerance to water deficiency and osmotic stress.

**Figure 3:**
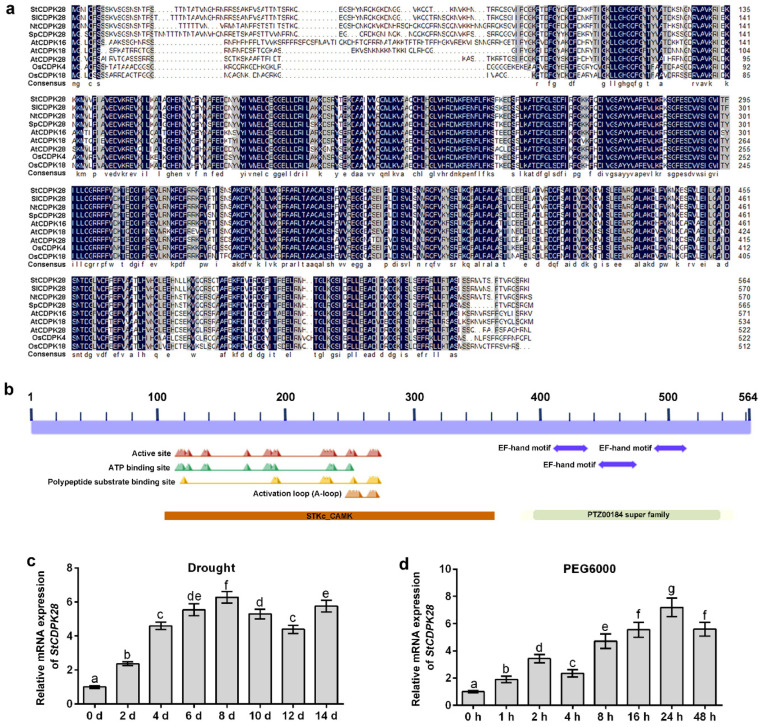
Identification of *CDPK28* induced by water deficiency and osmotic stress in potato. (**a**) Multiple sequence alignment of CDPKs. (**b**) Schematic diagram of the domain structures of StCDPK28 protein. (**c**) Drought. (**d**) PEG6000. The letters indicate significant differences according to one-way ANOVA corrected by Dunnett (*p* < 0.05).

The CDPK28-like protein in potato was predicted to encode a calcium-dependent protein kinase with 564 amino acids (sequence ID: XP_006340738.1). According to results of protein sequence matches with the basic local alignment search tool (BLAST) (https://blast.ncbi.nlm.nih.gov/Blast.cgi?PROGRAM=blastp&PAGE_TYPE=BlastSearch&LINK_LOC=blasthome, accessed on 27 September 2021), StCDPK28 protein showed sequence homology to SlCDPK28, NtCDPK28, SpCDPK28, AtCDPK16, AtCDPK18, AtCDPK28, OsCDPK4 and OsCDPK18, as depicted in Figure 3a. The main domain structures of StCDPK28 were mapped with the conserved domains search (CD-Search) tool (https://www.ncbi.nlm.nih.gov/Structure/cdd/wrpsb.cgi, accessed on 27 September 2021), containing the active site, ATP binding site, polypeptide substrate binding site, activation loop and EF-hand motif, as well as the STKc_CAMK domain (the catalytic domain of the CAMPK family serine/threonine kinases; STKc transfers the γ-phosphoryl group from ATP to serine/threonine residues) and the PTZ00184 super family domain (calmodulin) (Figure 3b). These results indicate that StCDPK28 plays a role as a calcium-dependent protein kinase.

### 2.2. Subcellular Localization of the StCDPK28 Protein and Construction of Potato Plants with Over- or Under-Expression of StCDPK28

**Figure 4:**
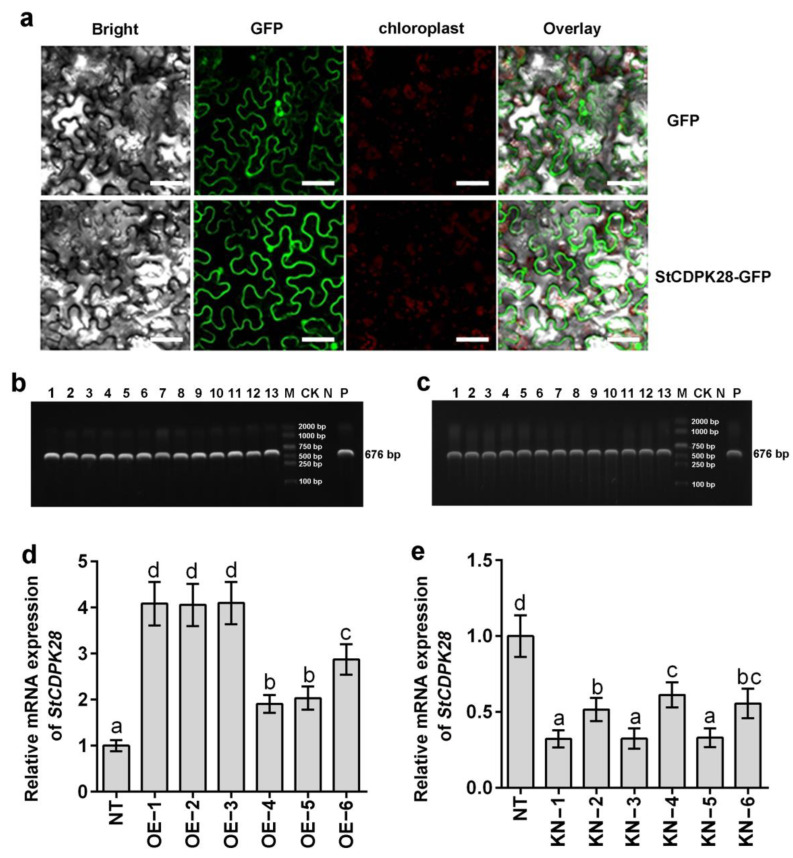
Subcellular localization of the StCDPK28-GFP fusion protein and establishment of transgenic potato plants overexpressing or underexpressing *StCDPK28*. (**a**) Confocal microscopy pictures of *Nicotiana tabacum* cells transformed with a *GFP*-*StCDPK28* construct using GFP as a control. Scale bars = 75 μm. PCR products of *NPTII* (676 bp) showing the introduction of (**b**) *StCDPK28* sequence into potato plant, as well as (**c**) amiStCDPK28 for *StCDPK28* knockdown. (**d**,**e**) Expression profiling of *StCDPK28* in 4-week-old potato leaves was analyzed by qRT-PCR with *StEf1a* as an internal control. Data are presented as the means ± standard deviation of nine replicates. The letters indicate significant differences according to one-way ANOVA corrected by Dunnett (*p* < 0.05).

To better understand the function of the StCDPK28 protein in the defense against water deficiency and osmotic stress, we investigated the subcellular localization of the StCDPK28 protein. An in vivo study was conducted in *Nicotiana tabacum* leaves. The results in Figure 4a showed that green fluorescence, generated by the recombinant pCAM35s-GFP-StCDPK28 construct, was strongly observed in the membrane, cytoplasm and nucleus.

To determine whether *StCDPK28* plays a role in the tolerance to water deficiency and osmotic stress, transgenic plants overexpressing *StCDPK28* (Figure 4b), and pCPI121-miRcdpk28 transformants were developed (Figure 4c). *StCDPK28*-overexpressing plants (OE-1, OE-2, OE-3, OE-4, OE-5, and OE-6) showed a relatively strong expression of *StCDPK28* (*p* < 0.05) (Figure 4d), and *StCDPK28*-knockdown plants (KD-1, KD-2, KD-3, KD-4, KD-5, and KD-6) showed a weak expression of *StCDPK28* (*p* < 0.05) (Figure 4e).

### 2.3. StCDPK28 Was Responsive to Water Deficiency and Osmotic Stress in Photosynthesis

**Figure 5:**
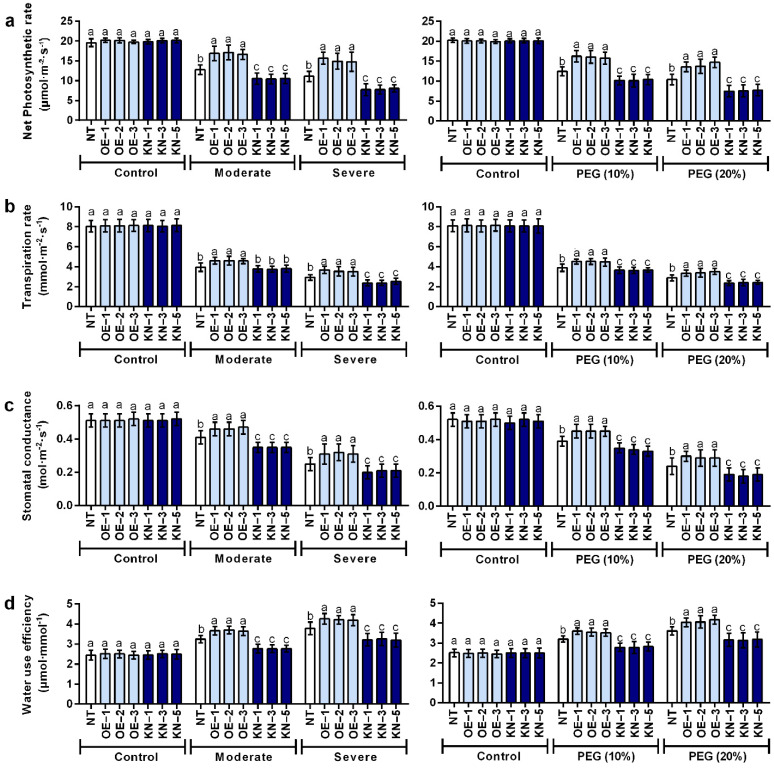
*StCDPK28* overexpression in potato enhances photosynthesis, transpiration, stomatal conductance and water use efficiency in response to water deficiency and osmotic stress. After the plants were cultivated under water deficiency and osmotic stress for 24 h, (**a**) net photosynthetic rate, (**b**) transpiration rate, (**c**) stomatal conductance, and (**d**) water use efficiency were evaluated. Data are presented as the means ± standard deviation of nine replicates. The letters indicate significant difference according to one-way ANOVA corrected by Dunnett (*p* < 0.05).

As for photosynthesis, we took into account the net photosynthetic rate, transpiration rate, stomatal conductance and water use efficiency. It was noticed that increases in net photosynthetic rate and transpiration were detected phenomenon induced by *StCDPK28* overexpression under water deficiency and osmotic stress (*p* < 0.05); however, a reverse result was observed in *StCDPK28*-knockdown plants (*p* < 0.05) (Figure 5a,b). The value of the stomatal conductance apparently increased in *StCDPK28*-overexpressing plants while dropped in *StCDPK28*-knockdown plants under water deficiency and osmotic stress (*p* < 0.05) (Figure 5c). Next, the water use efficiency was analyzed under water deficiency and osmotic stress. It was noticed that *StCDPK28*-overexpressing plants responded to water deficiency and osmotic stress with water use efficiency being increased (*p* < 0.05), while for *StCDPK28*-knockdown plants, water use efficiency dropped (*p* < 0.05) (Figure 5d). Interestingly, there was no obvious changes in the net photosynthetic rate, transpiration rate, stomatal conductance or water use efficiency of *StCDPK28*-overexpressing and *StCDPK28*-knockdown plants when compared to the NT under normal conditions (*p* > 0.05) (Figure 5a–d).

### 2.4. StCDPK28 Modulated the Contents of H_2_O_2_, Malondialdehyde (MDA), and Proline for Tolerance to Water Deficiency and Osmotic Stress

**Figure 6:**
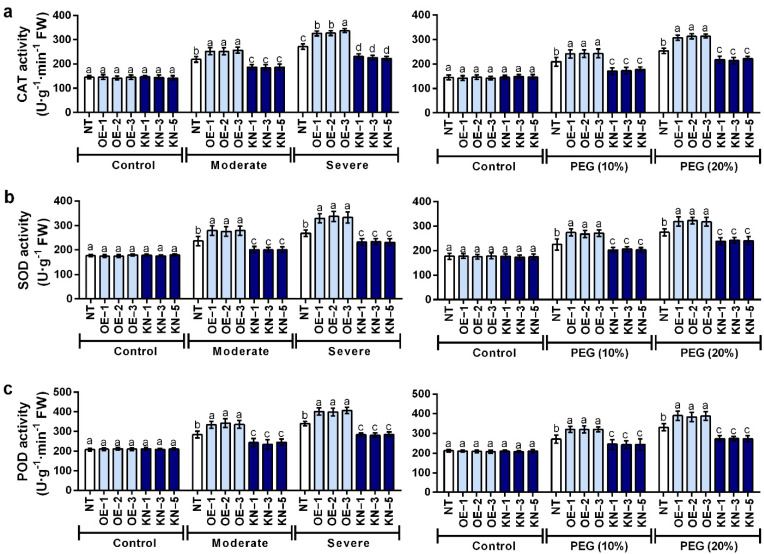
Upregulation of *StCDPK28* counteracts the effects of water deficiency and PEG stress on the contents of H_2_O_2_, MDA and proline. Contents of (**a**) H_2_O_2_, (**b**) MDA, and (**c**) proline were detected 24 h after water deficiency and PEG treatment. Data are presented as the means ± standard deviation of nine replicates. The letters indicate significant difference according to one-way ANOVA corrected by Dunnett (*p* < 0.05).

Water deficiency and osmotic stress significantly decreased the H_2_O_2_ content in *StCDPK28*-overexpressing plants with respect to the normal plants (*p* < 0.05), while the H_2_O_2_ content in *StCDPK28*-knockdown plants were not obviously changed under moderate water deficiency and osmotic stress (*p* > 0.05), and only severe water deficiency elevated the H_2_O_2_ content in *StCDPK28*-knockdown plants (*p* < 0.05) (Figure 6a). MDA, as an essential indicator of plant resistance to water deficiency and osmotic stress, was reduced in *StCDPK28*-overexpressing plants exposed to water deficiency treatment (moderate and severe) and 20% PEG6000 (*p* < 0.05); as for *StCDPK28*-knockdown plants, an elevation in MDA content was noticed (*p* < 0.05) (Figure 6b). Proline content was evidently increased by *StCDPK28* overexpression in response to water deficiency and osmotic stress (*p* < 0.05); however, the decrement of proline content occurred in *StCDPK28*-knockdown plants (*p* < 0.05) (Figure 6c). Consistent with the results of photosynthesis, it was found that *StCDPK28* overexpression and knockdown had no major role in the adaptation of potato plants under normal conditions (*p* > 0.05) (Figure 6a–c).

### 2.5. Catalase (CAT), Superoxide Dismutase (SOD) and Peroxidase (POD) Activities in StCDPK28-Overexpressing and StCDPK28-KnockDown Plants under Water Deficiency and Osmotic Stress

**Figure 7:**
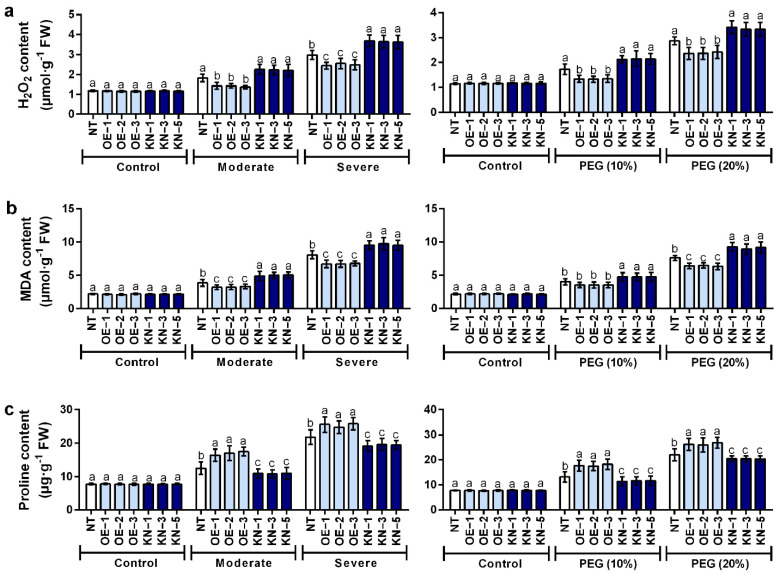
Activity of CAT, SOD and POD increased by water deficiency and osmotic stress is positively regulated by *StCDPK28* overexpression. Activity of (**a**) CAT, (**b**) SOD and (**c**) POD was examined 24 h after potato plants were treated by water deficiency and PEG. Data are presented as the means ± standard deviation of nine replicates. The letters indicate significant difference according to one-way ANOVA corrected by Dunnett (*p* < 0.05).

*StCDPK28* overexpression increased CAT activity, while *StCDPK28* knockdown resulted in a marked decline in CAT activity under moderate and severe water deficiency conditions, as well as osmotic stress conditions (10% and 20% PEG) (*p* < 0.05) (Figure 7a). Similarly, a significant enhancement in SOD activity was detected in potato plants overexpressing *StCDPK28* after cultivation under water deficiency conditions or osmotic stress (*p* < 0.05); on the contrary, a significant decrease in SOD activity was noticed in *StCDPK28*-knockdown plants (*p* < 0.05) (Figure 7b). Regarding POD activity, a positive effect of *StCDPK28* overexpression occurred (*p* < 0.05), and a negative effect of *StCDPK28*-knockdown was recorded (*p* < 0.05) in response to water deficiency and osmotic stress (Figure 7c). However, a non-significant effect of *StCDPK28* overexpression or knockdown was exhibited on CAT, SOD and POD activity under normal conditions (*p* > 0.05) (Figure 7a–c).

### 2.6. Sequence Accession Numbers

Sequence information presented in this article can be obtained in the protein libraries using the accession code: StCDPK28, XP_006340738.1; SlCDPK28, XP_004232492.1; NtCDPK28, XP_016507827.1; SpCDPK28, XP_015070510.1; AtCDPK16, NP_179379.1; AtCDPK18, NP_195331.2; AtCDPK28, NP_851280.1; OsCDPK4, BAG87555.1; OsCDPK18, BAH00509.1.

## 3. Discussion

Plant growth is influenced by multiple exogenous elements and this is strongly linked to a sophisticated interplay of stress-associated kinases [25,26]. At present, we have provided experimental evidence that water deficiency and osmotic stress altered the expression patterns of *StCDPKs*. *StCDPK28* was defined as a positive modulator of plant stress responses. Water deficiency and osmotic stress induced *StCDPK28* overexpression in the potato plant. *StCDPK28* conferred no significant effects on physiological activities under normal conditions. However, we suggested that *StCDPK28* was a critical modulatory kinase implicated in water deficiency and the osmotic stress response by enhancing the physiological activities.

Phylogenetic analysis revealed that *CDPK28* of the potato plant was orthologous to *SlCDPK28*, *NtCDPK28*, *SpCDPK28*, *AtCDPK16*, *AtCDPK18*, *AtCDPK28*, *OsCDPK4* and *OsCDPK18*, followed by identification of *StCDPK28* via sequence alignment in this study. Besides, transient expression analysis revealed that StCDPK28 exhibited a membrane, cytosolic and nuclear localization. Studies have reported that potato plants establish their defense mechanisms by changing their *CDPKs* expression levels in response to environmental stress, including biotic and abiotic factors [15,27]. *Phytophthora infestans* causes systemic *StCDPK7* overexpression in the leaves of potato plant [15]. *StCDPK2* expression is highly induced by light treatment, which is associated with its light-responsive, *cis*-acting elements [27]. Our work showed that *StCDPK28* displayed an expression increment in potato leaves, which was caused by water deficiency and osmotic stress. Furthermore, several studies reported that CDPK28 kinase activity strictly depends on calcium which modulates CDPK28 phosphorylation [18,20]. Together, upregulation of *StCDPK28* by water deficiency and osmotic stress might function in response to adverse conditions, in which the Ca^2+^ messenger is involved.

Under normal circumstances, *CDPK28* modulates phenotypic changes in lignin deposition [17], morphology [19], plant stem elongation and vascular development [20], as well as regulating the immune signaling [21,22] of *Arabidopsis thaliana*. In this study, we observed that the photosynthetic activity of *StCDPK28*-overexpressing or *StCDPK28*-knockdown plants was not significantly changed, in addition to the contents of H_2_O_2_, MDA and proline, and the activities of CAT, SOD and POD. A mechanism, previously described for *StCDPK28*, in which the introduction of autophosphorylation contributes to constitutive kinase activity, can be included for *StCDPK28* [18]. The activity of Ca^2+^-responsive StCDPK28 might be not changed in *StCDPK28*-overexpressing or *StCDPK28*-knockdown potato plants because of the unchanged physiological concentration of Ca^2+^. However, complementary experiments are required to prove the precise mechanism.

Interestingly, combined with wild-type plants after water deficiency and osmotic stress treatment, transgenic lines with four- to five-fold higher *StCDPK28* expression levels showed an obvious overexpression phenotype, such as increased photosynthetic activity, contents of H_2_O_2_, MDA and proline, as well as activities of CAT, SOD and POD. Likewise, physiologic property changes induced by loss-of-function were observed in any of the plant lines that expressed low levels of *StCDPK28* in response to water deficiency and osmotic stress. Calcium is one of the well-documented signaling components regulating the abiotic and biotic stress signaling mechanism in plants [26]. Calcium-dependent protein kinases dominate the calcium-dependent stress signaling mechanisms in response to drought, osmotic and other environmental stressors [28]. Drought decreases water potential and photosynthetic activity [29]. Besides, drought-induced conformational changes such as abscisic-acid-dependent stomatal movement, osmotic adjustments and oxidative damage [30]. Water deficiency provokes the changes of cytosolic Ca^2+^ concentration, and then activates CDPK that induces the release of abscisic acid [31]. Hence, our results suggest that *StCDPK28* might be upregulated in potato for maintaining physiological properties in response to water deficiency and osmotic stress.

## 4. Materials and Methods

### 4.1. Plasmid Construction and Transformation

Whole RNA from the leaves of potato cv. Zihuabai was extracted using the TRIzol RNA Extraction Kit (Invitrogen, Carlsbad, CA, USA). The cDNA was synthesized using the First-Strand cDNA Synthesis Kit (TransGen Biotech, Beijing, China), according to the user’s manual. The generated cDNA was used as the template for *StCDPK28* (GenBank Accession No. XM_006340676.2) amplification with the specific primers (forward primer 5′-GCTCTAGACCTTTCTTCCTCCTCCTATTTC-3′ carrying *Xba* I site [5′-GCTCTAGA-3′] and reverse primer 5′-CGGGATCCGCTGGTTGTTTAGGAGAAAGC-3′ carrying *Bam*H I site [5′-CGGGATCC-3′]), and then the *StCDPK28* sequence was infused into a pCPB plasmid (pCPB-StCDPK28). A microRNA sequence targeting *StCDPK28* mRNA was generated with the designed primers (miR-s 5′- GATATTGTAATGAGACCCCGCTTTCTCTCTTTTGTATTCC-3′, miR-a 5′-GAA AGCGGGGTCTCATTACAATATCAAAGAGAATCAATGA-3′, miR-s* 5′-GAAAA CGGGGTCTCAATACAATTTCACAGGTCGTGATATG-3′, and miR-a* 5′-GAA ATTGTATTGAGACCCCGTTTTCTACATATATATTCCT-3′), and the generated miRcdpk28 sequence (5′-TATTGTAATGAGACCCCGCTT-3′) was ligated into the pCPBI121 plasmid (pCPBI121-miRcdpk28), with reference to Zhou’s description [32]. *Agrobacterium tumefaciens* LBA4404 were transformed with pCPB-StCDPK28 or pCPBI121-miRcdpk28, and transgenic potato plants were developed by following the methods of Si et al. [33]. In short, potato tubers with a diameter of 0.5 cm and a thickness of 2 mm were infected with the inoculum of LBA4404 for 10 min, followed by cultivation in MS media containing 3% sucrose under an 8-h dark period (15 °C) and 16-h photoperiod (22 °C, 3500 Lx) in Biotron at 22 °C. After 4 weeks, the transgenic plants were collected by incubation in 75 mg/L kanamycin, which were confirmed by PCR, as described below.

### 4.2. Water Deficiency and Osmotic Stress Treatment

To analyze the effects of water deficiency and osmotic stress on *StCDPK* expression, 4-week-old potato plantlets were continually cultivated in an MS medium supplemented with 8% sucrose in the dark. After 30 days, the generated potato tubers were collected, and the sprouting tubers were transferred into pots (height: 11.2 cm, width: 16.8 cm) filled with soil and vermiculite (1:1, *v*/*v*). The sprouting tubers were cultured for 30 days. For the water deficiency treatment, the plants were cultivated without irrigation, and the leaves were analyzed on day 0, 1, 2, 3, 4, 5 and 6, for *StCDPKs* expression by qRT-PCR; for osmotic stress, *StCDPK* was analyzed in leaves of 20% PEG-treated potato plants that were collected 0, 1, 2, 4, 8, 16 and 24 h after treatment. The sprouting tubers generated from the wild type and transgenic potato plantlets (4 weeks old) were cultured for 30 days, and allocated to the type of water deficiency treatment, that is, control (75–80% soil water content), moderate water deficiency (55–60% soil water content) and severe water deficiency (30–35% soil water content). As for PEG treatment, potato plants were treated with or without PEG (10% and 20%), respectively. Potato plants were subjected to downstream analysis 24 h after treatment.

### 4.3. Validation of the Transgenic Potato Plants by PCR

The cDNA from the transgenic and non-transgenic plants was subjected to PCR assay for the *NPTII* maker. PCR was carried out using the TakaRa Ex Taq Kit (Takara, Tokyo, Japan) according to the supplier’s instruction. PCR conditions were 30 cycles of denaturation for 30 sec at 94 °C, annealing for 30 sec at 55 °C and extension for 1 min at 72 °C. The constructed plasmids (pCPB-StCDPK28 and pCPBI121-miRcdpk28) were assayed for *NPTII*, and served as a positive control (P). Potato plants without transfection served as the control (CK). The ddH_2_O served as negative control (N). The sequence of the primers was as follows: *NPTII* forward primer 5′-GCTATGACTGG GCACAACAG-3′ and reverse primer 5′-ATACCGTAAAGCACGAGGAA-3′.

### 4.4. Quantification of Gene Expression by qRT-PCR

To investigate relative mRNA levels of *StCDPKs*, cDNA generated from the whole RNA in plant leaves was used for the qRT-PCR analysis. A reaction mixture for qPCR consisted of 100 ng of cDNA, 0.6 μL of specific primers (10 μM), 10 μL of 2× SuperReal PreMix Plus and 0.4 μL of 50× ROX Reference Dye (Tiangen Biotech, Beijing, China) to a final volume of 20 μL. The thermal profile of the ABI3000 system (Applied Biosystems, Foster City, CA, USA) were as follows: initial denaturation for 2 min at 94 °C, and 40 cycles of denaturation for 30 s at 94 °C, annealing for 34 s at 60 °C and extension for 30 s at 72 °C. The cycle threshold (CT) values were obtained and the mRNA level was calculated based on formula 2^−ΔΔCt^. Each experiment was carried out in three technical and three biological replicates. *StEf1a* was used as an internal control. Primer sequences were referenced from our previous study [34].

### 4.5. Subcellular Location of StCDPK28

To investigate the subcellular localization of the StCDPK28 protein, a full sequence of *StCDPK28* was ligated into pCAM35s-GFP. The plasmid was provided by Bioediates (Shanxi, China). The empty vector (pCAMP3s-GFP) served as a localization control. For agroinfiltration, the mixture of Gv3101 supernatant (OD_600_ = 0.4, 125 μL) and infiltration media (375 μL) were used to infiltrate three-week-old *Nicotiana tabacum* plants according a previous method [35]. The agroinfiltrated plants were kept at 24 °C for 2 days in the dark. The segments (2 cm^2^) were excised from the infiltrated zone of the leaf tissues, followed by observation under a Leica TCA confocal microscope (Leica, Weztlar, Germany).

### 4.6. Photosynthesis, Transpiration, Stomatal Conductance and Water Use Efficiency

To determine photosynthesis and transpiration, a portable photosynthetic system LI-6400XT (Li-COR, Lincoln, NE, USA) was used. The determination was performed on the second fully expanded leaf which was counted from the apex. Light function was examined at an irradiance of 1500 µmol·m^−2^·s^−1^. The concentration of CO_2_ was controlled by the facility at 400 µmol·mol^−1^. Water use efficiency was defined as the ratio of the net photosynthesis rate to transpiration rate.

### 4.7. CAT, SOD and POD Activity

The fifth or sixth leaf below the growing point was selected for the determination of CAT, SOD and POD activity. Fresh leaves (0.5 g) were ground in a pre-cold potassium phosphate buffer (pH 7.0), followed by transference into a 25 mL volumetric flask. The extraction was maintained at 5 °C for 10 min. The supernatant was collected and then centrifuged at 4000 rpm for 15 min. CAT, SOD and POD activity was examined as described previously [36], which was presented in our previous study [34].

### 4.8. H_2_O_2_, MDA and Proline Contents

H_2_O_2_ contents were determined according to a previous method [37] with minor modification. In brief, 0.5 g of leaves were extracted with 5 mL of TCA (0.1%, *w*/*v*), and the extract was subjected to centrifugation (12,000 rpm, 15 min). The supernatant (0.5 mL) was then collected and diluted with 1 mol/L KI and 0.5 mL of potassium phosphate buffer (10 mM, pH 7.0). The absorbance was detected spectrophotometrically at 390 nm.

In terms of MDA content, Heath’s method was referenced [38] with minor modification. In short, 0.2 g of fresh leaves were extracted with 5 mL of TCA (10%). The extract was centrifuged at 4000× *g* for 10 min, and the supernatant was gathered. The supernatant (2 mL) was mixed with 2 mL of 0.6% TBA prepared in 10% TCA, followed by incubation at 100 °C for 15 min. After centrifugation (3500 rpm, 10 min), the absorbance at 532 nm, 600 nm and 450 nm was determined.

Proline contents were examined according to the method of Bates [39] with minor modification. Shortly after, potato leaves (0.2 g) were homogenized in 5 mL of 3% sulfosalicylic acid and the mixture was maintained in a boiling water bath for 10 min. After cooling, 2 mL of the supernatant was added with 3 mL of 2.5% ninhydrin and 2 mL of acetic acid. The color reaction continued for 40 min in a boiling water bath. The product was extracted with toluene, and the absorbance was determined at 520 nm.

### 4.9. Statistical Analysis

Three biological and technical replicates were conducted in each experiment. Statistical analysis was performed using IBM SPSS 19.0 statistical software (IBM, Chicago, IL, USA). Multiple comparisons were carried out using one-way ANOVA corrected by Dunnett. The obtained data were presented as the mean ± standard deviations. *p*-values less than 0.05 indicated the statistical difference.

## 5. Conclusions

Taken together, the altered expression profiles of *StCDPK* suggested that *StCDPKs* were involved in the response of the potato plant to water deficiency and osmotic stress. StCDPK28, as a calcium-dependent protein kinase, showed water-deficiency- and osmotic-stress-responsive features. In potato plants, StCDPK28 was localized in the membrane, cytoplasm and nucleus. This study demonstrated the modulatory roles of *StCDPK28* in photosynthetic and physiological activities in response to water deficiency and osmotic stress. Further work will definitely be required to investigate the downstream targets of *StCDPK28*, which may decipher the underlying mechanisms whereby *StCDPK28* mediates the responsiveness of potato plants in terms of water deficiency and osmotic stress. Regulation of StCDPK28 expression could be a promising approach to improve the tolerance ability of potato plants in response to water deficiency or high salt media. The findings may provide a framework for implementing water-saving deficit irrigation under different local soil and climatic conditions.

## Data Availability

The datasets used and/or analyzed during the current study are available from the corresponding author on reasonable request.

## References

[1] Jansky S.H., Jin L.P., Xie K.Y., Xie C.H., Spooner D.M. (2009). Potato production and breeding in China. Potato Res..

[2] Kumar D., Minhas J., Singh B., Paul Khurana S.M., Minhas J.S., Pandey S.K. (2003). Abiotic stress and potato production. The Potato: Production and Utilization in Sub-Tropics.

[3] Obidiegwu J.E., Bryan G.J., Jones H.G., Prashar A. (2015). Coping with drought: Stress and adaptive responses in potato and perspectives for improvement. Front. Plant Sci..

[4] Handayani T., Gilani S.A., Watanabe K.N. (2019). Climatic changes and potatoes: How can we cope with the abiotic stresses?. Breed. Sci..

[5] Ruttanaprasert R., Jogloy S., Vorasoot N., Kesmala T., Kanwar R.S., Holbrook C.C., Patanothai A. (2016). Effects of water stress on total biomass, tuber yield, harvest index and water use efficiency in Jerusalem artichoke. Agric. Water Manag..

[6] Muñiz García M.N., Cortelezzi J.I., Fumagalli M., Capiati D.A. (2018). Expression of the Arabidopsis *ABF4* gene in potato increases tuber yield, improves tuber quality and enhances salt and drought tolerance. Plant Mol. Biol..

[7] Liese A., Romeis T. (2013). Biochemical regulation of in vivo function of plant calcium-dependent protein kinases (CDPK). Biochim. Biophys. Acta Mol. Cell Res..

[8] Shi S., Li S., Asim M., Mao J., Xu D., Ullah Z., Liu G., Wang Q., Liu H. (2018). The Arabidopsis calcium-dependent protein kinases (CDPKs) and their roles in plant growth regulation and abiotic stress responses. Int. J. Mol. Sci..

[9] Cui X.-Y., Du Y.-T., Fu J.-d., Yu T.-F., Wang C.-T., Chen M., Chen J., Ma Y.-Z., Xu Z.-S. (2018). Wheat CBL-interacting protein kinase 23 positively regulates drought stress and ABA responses. BMC Plant Biol..

[10] Chen X., Ding Y., Yang Y., Song C., Wang B., Yang S., Guo Y., Gong Z. (2021). Protein kinases in plant responses to drought, salt, and cold stress. J. Integr. Plant Biol..

[11] Klimecka M., Muszyńska G. (2007). Structure and functions of plant calcium-dependent protein kinases. Acta Biochim. Pol..

[12] Tuteja N., Mahajan S. (2007). Calcium signaling network in plants: An overview. Plant Signal. Behav..

[13] Asano T., Hakata M., Nakamura H., Aoki N., Komatsu S., Ichikawa H., Hirochika H., Ohsugi R. (2011). Functional characterisation of OsCPK21, a calcium-dependent protein kinase that confers salt tolerance in rice. Plant Mol. Biol..

[14] Asano T., Hayashi N., Kobayashi M., Aoki N., Miyao A., Mitsuhara I., Ichikawa H., Komatsu S., Hirochika H., Kikuchi S. (2012). A rice calcium-dependent protein kinase OsCPK12 oppositely modulates salt-stress tolerance and blast disease resistance. Plant J..

[15] Fantino E., Segretin M.E., Santin F., Mirkin F.G., Ulloa R.M. (2017). Analysis of the potato calcium-dependent protein kinase family and characterization of StCDPK7, a member induced upon infection with *Phytophthora infestans*. Plant Cell Rep..

[16] Santin F., Bhogale S., Fantino E., Grandellis C., Banerjee A.K., Ulloa R.M. (2017). *Solanum tuberosum* StCDPK1 is regulated by miR390 at the posttranscriptional level and phosphorylates the auxin efflux carrier StPIN4 in vitro, a potential downstream target in potato development. Physiol. Plant..

[17] Jin Y., Ye N., Zhu F., Li H., Wang J., Jiang L., Zhang J. (2017). Calcium-dependent protein kinase CPK28 targets the methionine adenosyltransferases for degradation by the 26S proteasome and affects ethylene biosynthesis and lignin deposition in Arabidopsis. Plant J..

[18] Bender K.W., Blackburn R.K., Monaghan J., Derbyshire P., Menke F.L., Zipfel C., Goshe M.B., Zielinski R.E., Huber S.C. (2017). Autophosphorylation-based calcium (Ca^2+^) sensitivity priming and Ca^2+^/calmodulin inhibition of *Arabidopsis thaliana* Ca(2+)-dependent protein kinase 28 (CPK28). J. Biol. Chem..

[19] Matschi S., Hake K., Herde M., Hause B., Romeis T. (2015). The calcium-dependent protein kinase CPK28 regulates development by inducing growth phase-specific, spatially restricted alterations in jasmonic acid levels independent of defense responses in Arabidopsis. Plant Cell.

[20] Matschi S., Werner S., Schulze W.X., Legen J., Hilger H.H., Romeis T. (2013). Function of calcium-dependent protein kinase CPK28 of *Arabidopsis thaliana* in plant stem elongation and vascular development. Plant J..

[21] Monaghan J., Matschi S., Romeis T., Zipfel C. (2015). The calcium-dependent protein kinase CPK28 negatively regulates the BIK1-mediated PAMP-induced calcium burst. Plant Signal. Behav..

[22] Monaghan J., Matschi S., Shorinola O., Rovenich H., Matei A., Segonzac C., Malinovsky F.G., Rathjen J.P., MacLean D., Romeis T. (2014). The calcium-dependent protein kinase CPK28 buffers plant immunity and regulates BIK1 turnover. Cell Host Microbe.

[23] Gao A., Wu Q., Zhang Y., Miao Y., Song C. (2014). Arabidopsis calcium-dependent protein kinase CPK28 is potentially involved in the response to osmotic stress. Chin. Sci. Bull..

[24] Ding Y., Yang H., Wu S., Fu D., Li M., Gong Z., Yang S. (2022). CPK28-NLP7 module integrates cold-induced Ca2+ signal and transcriptional reprogramming in Arabidopsis. Sci. Adv..

[25] Zhu J.K. (2016). Abiotic stress signaling and responses in plants. Cell.

[26] Ranty B., Aldon D., Cotelle V., Galaud J.P., Thuleau P., Mazars C. (2016). Calcium sensors as key hubs in plant responses to biotic and abiotic stresses. Front. Plant Sci..

[27] Giammaria V., Grandellis C., Bachmann S., Gargantini P.R., Feingold S.E., Bryan G., Ulloa R.M. (2011). StCDPK2 expression and activity reveal a highly responsive potato calcium-dependent protein kinase involved in light signalling. Planta.

[28] Schulz P., Herde M., Romeis T. (2013). Calcium-dependent protein kinases: Hubs in plant stress signaling and development. Plant Physiol..

[29] Zandalinas S.I., Mittler R., Balfagón D., Arbona V., Gómez-Cadenas A. (2018). Plant adaptations to the combination of drought and high temperatures. Physiol. Plant..

[30] Singh A., Sagar S., Biswas D.K. (2017). Calcium dependent protein kinase, a versatile player in plant stress management and development. Crit. Rev. Plant Sci..

[31] Zou J.J., Li X.D., Ratnasekera D., Wang C., Liu W.X., Song L.F., Zhang W.Z., Wu W.H. (2015). Arabidopsis CALCIUM-DEPENDENT PROTEIN KINASE8 and CATALASE3 function in abscisic acid-mediated signaling and H_2_O_2_ homeostasis in stomatal guard cells under drought stress. Plant Cell Rep..

[32] Zhou X., Zhang N., Yang J., Tang X., Wen Y., Si H. (2018). Functional analysis of StDWF4 gene in response to salt stress in potato. Plant Physiol. Biochem..

[33] Si H.J., Xie C.H., Liu J. (2003). An efficient protocol for *Agrobacterium*-mediated transformation with microtuber and the introduction of an antisense class I patatin gene into potato. Acta Agron. Sin..

[34] Zhu X., Hong X., Liu X., Li S., Yang J., Wang F., Yue Y., Zhang N., Si H. (2021). Calcium-dependent protein kinase 32 gene maintains photosynthesis and tolerance of potato in response to salt stress. Sci. Hortic..

[35] Sparkes I.A., Runions J., Kearns A., Hawes C. (2006). Rapid, transient expression of fluorescent fusion proteins in tobacco plants and generation of stably transformed plants. Nat. Protoc..

[36] Li H. (2000). Determination of superoxide dismutase activity by the means of nitroblue tetrazolium. Principles and Techniques of Plant Physiological Biochemical Experiments.

[37] Sergiev I., Alexieva V., Karanov E. (1997). Effect of spermine, atrazine and combination between them on some endogenous protective systems and stress markers in plants. C. R. Acad. Bulg. Sci..

[38] Heath R.L., Packer L. (1968). Photoperoxidation in isolated chloroplasts. I. Kinetics and stoichiometry of fatty acid peroxidation. Arch. Biochem. Biophys..

[39] Bates L.S., Waldren R.P., Teare I.D. (1973). Rapid determination of free proline for water-stress studies. Plant Soil.

